# Supplementary material for: Large influence of dust on the Precambrian climate
Source: Nat Commun. 2020 Sep 4;11:4427. doi: 10.1038/s41467-020-18258-2 (PMC7474055; doi:10.1038/s41467-020-18258-2)
Supplement: Supplementary file 1 — Supplementary Information [file 41467_2020_18258_MOESM1_ESM.pdf]

Supplementary Information for

Large Influence of Dust on the Precambrian Climate

Liu et al.

Supplementary Table 1. Summary of model results

Erodibility	Emissions Tg yr^{-1}	Loading Tg	Fraction of particles (0-2.5 µm) %	DOD	Temp. °C	Surface Albedo	Deposit in ocean Tg yr^{-1}	DDSE W/m2
0.0	NA	NA	NA	NA	14.5	0.16	NA	NA
0.0375	36,463	312	45.8	0.38	3.3	0.27	12,271	-22.5
0.0375 DV	13,516	132	46.9	0.17	9.8	0.21	5,159	-11.1
0.075	60,419	488	48.0	0.62	0.0	0.30	20,437	-32.4
0.075 DV	21,667	220	50.5	0.29	7.5	0.23	8,460	-17.4
0.15	85,698	689	51.8	0.92	-2.5	0.33	28,662	-41.9
0.15 DV	31,194	316	54.9	0.45	5.5	0.26	12,449	-23.9
0.3	132,303	1114	56.5	1.61	-5.4	0.37	44,780	-57.8
0.3 DV	45,982	471	61.7	0.72	2.6	0.29	16,834	-33.7
PI	2,365[1]	27.5[1]	NA	0.032[1]	13.8[2]	0.14[2]	1,275[3]	-2.0[4]

DV: simulation with dynamic vegetation turned on; PI: Pre-Industrial Conditions; DOD: Dust Optical Depth; DDSE: Dust Direct Shortwave Effect which represents the reduction of received surface solar radiation due to dust.

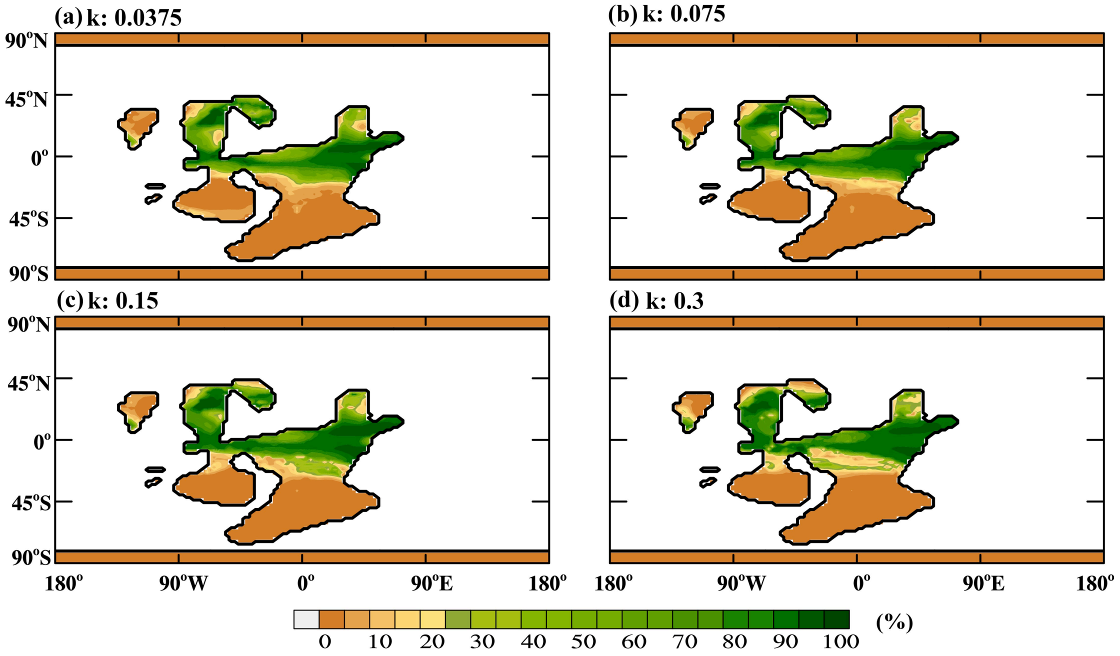

Supplementary Figure 1. Annual mean fraction of vegetated area at different surface erodibilities. The fraction is calculated for each grid box. All types of vegetation such as trees, shrubs and grass are included. Unit: %.

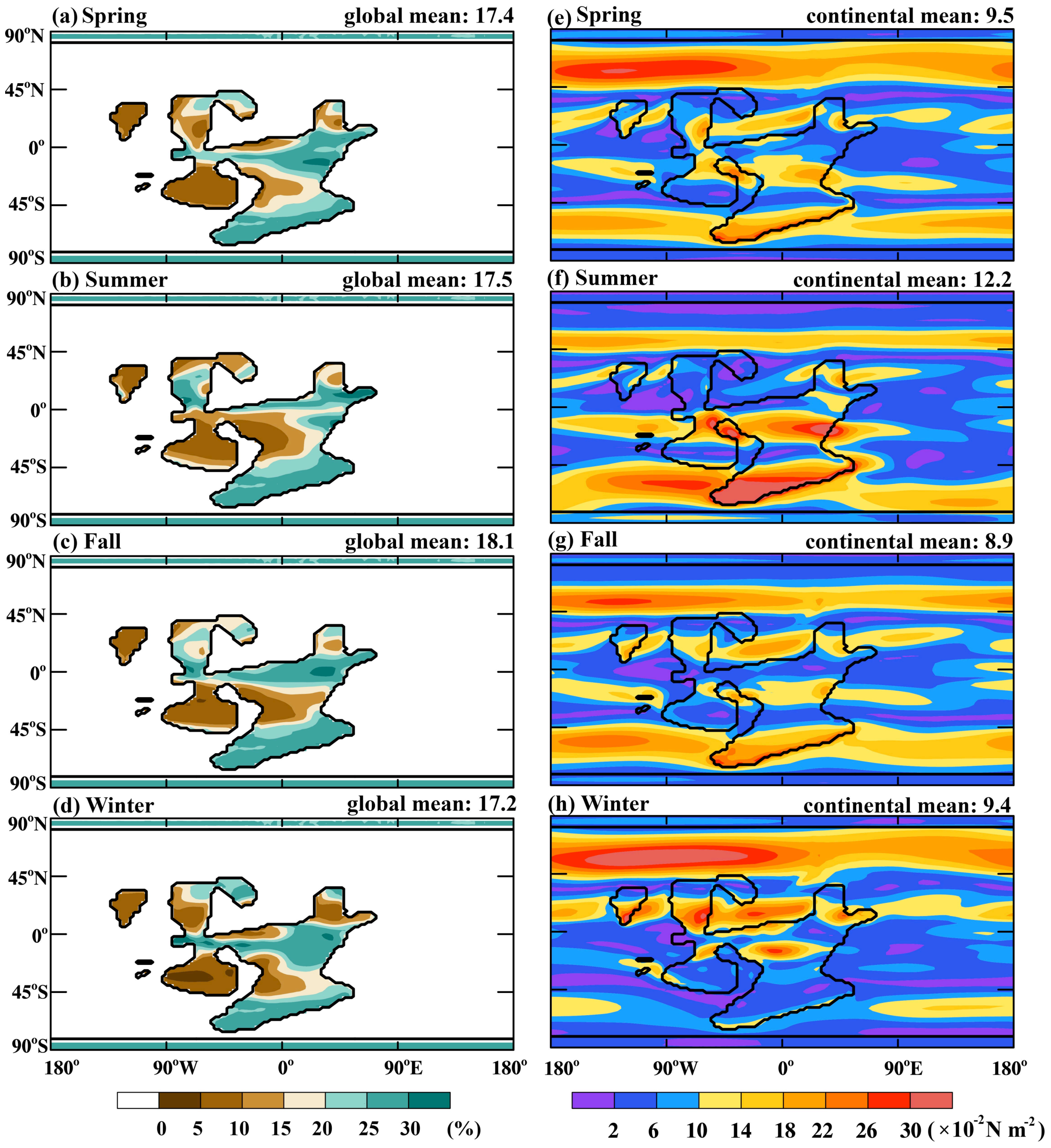

Supplementary Figure 2. Seasonal mean soil moisture and wind stress when surface erodibility is 0.0375 without vegetation. (a-d) Soil moisture for different seasons in unit: % by volume; (e-h) wind stress in unit: N m^{-2}. Each season has three consecutive months and the Spring starts from March.

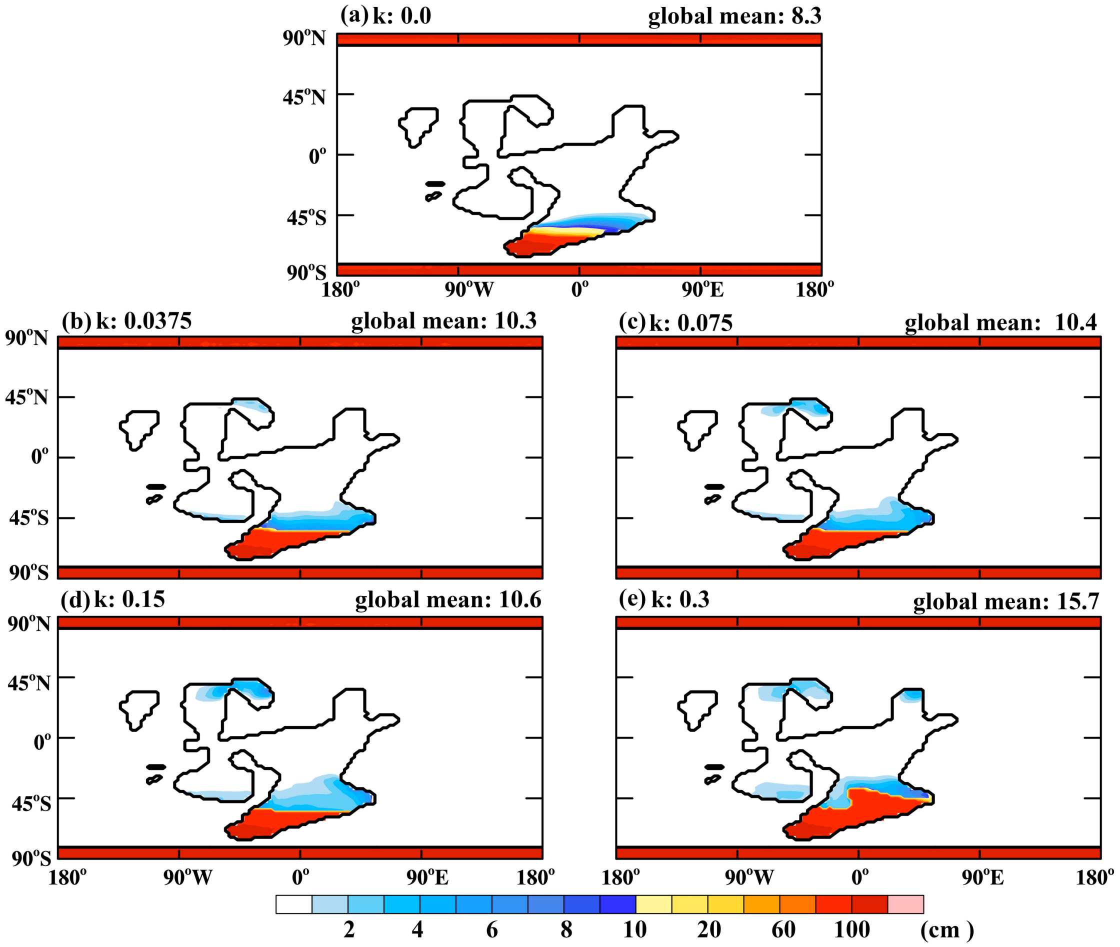

Supplementary Figure 3. Annual mean snow depth on land under different erodibilities when land vegetation is absent. Unit: cm.

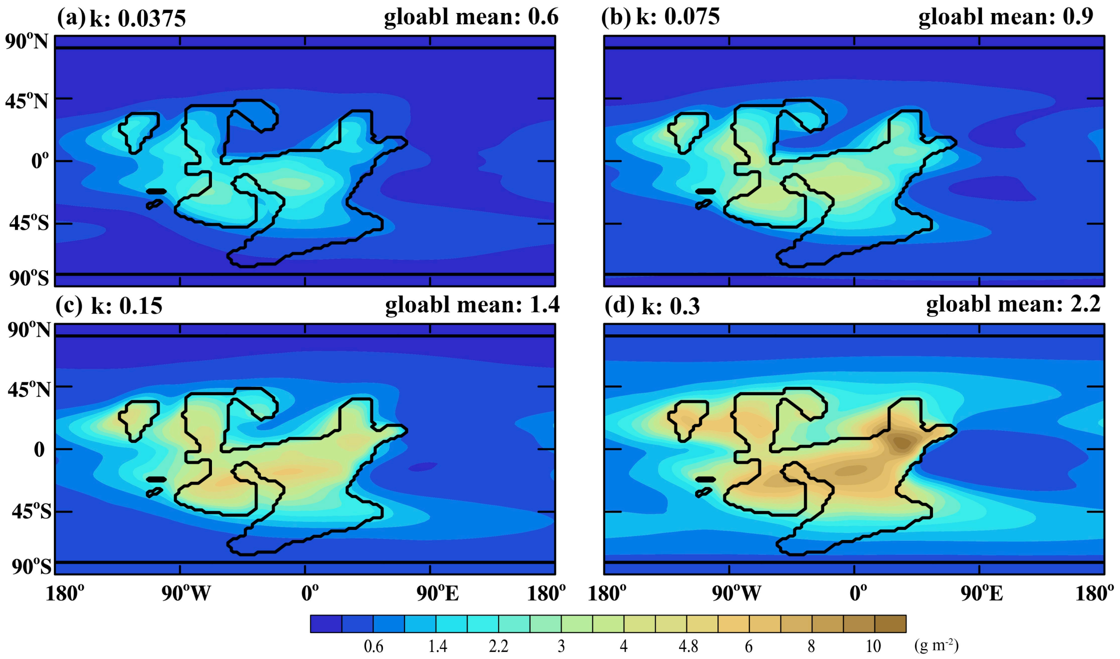

Supplementary Figure 4. Annual mean column integrated atmospheric dust loading under different erodibilities when land vegetation is absent. Unit: g m^{-2}.

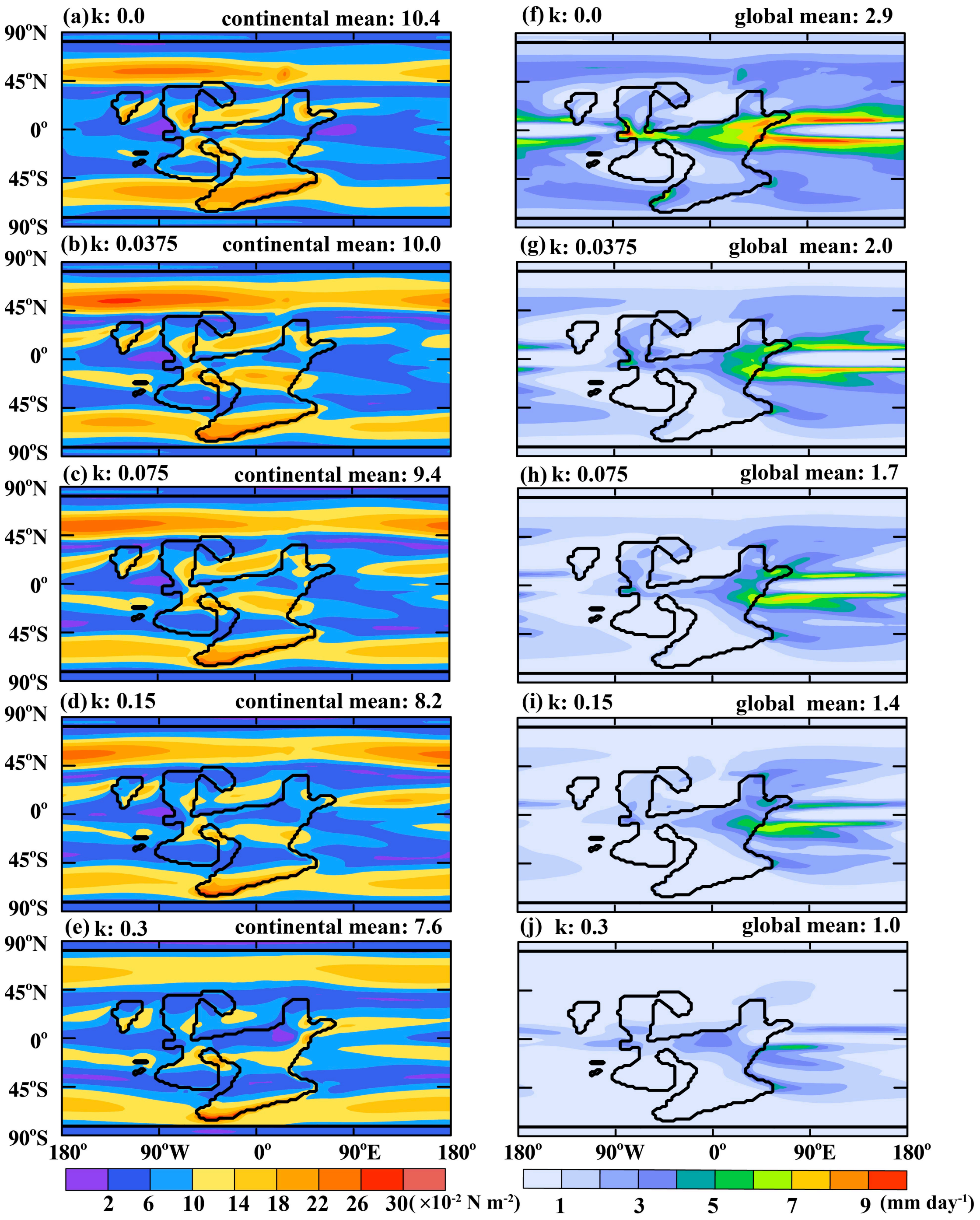

Supplementary Figure 5. Annual mean surface wind stress and precipitation under different erodibilities when land vegetation is absent. (a-e) the annual mean absolute wind stress in unit: N/m^2; (f-j) precipitation rate in unit: mm/day.

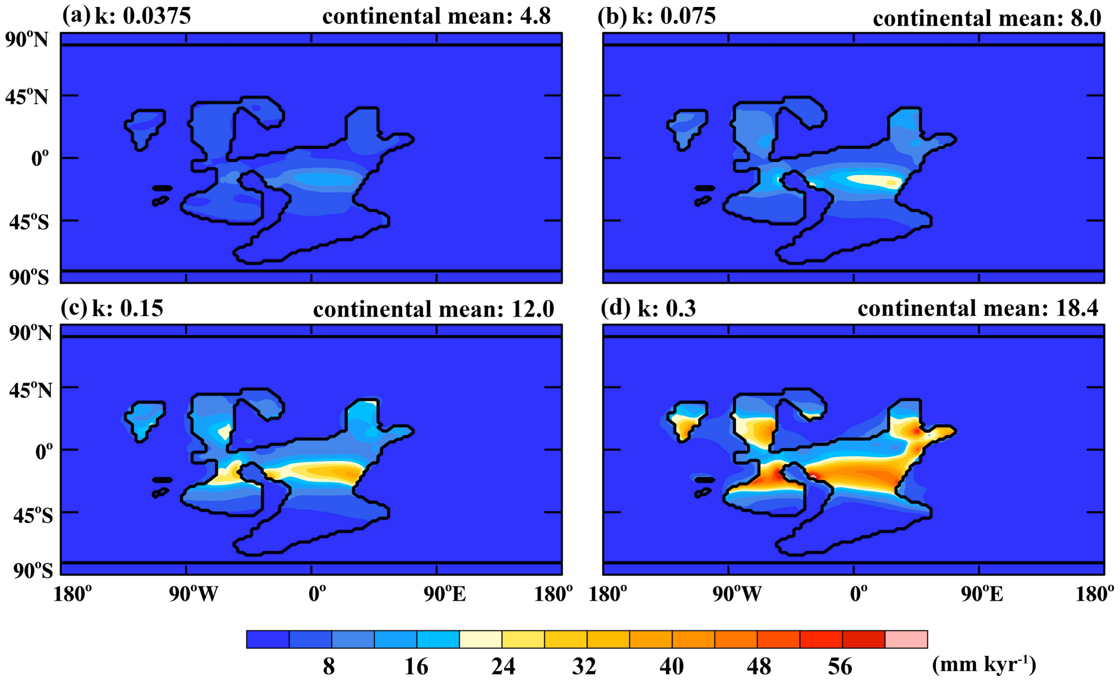

Supplementary Figure 6. Dust deposition rate under different erodibilities when land vegetation is absent. It is calculated assuming a density of 2500 kg m^{-3} for the dust. Unit: mm kyr^{-1}.

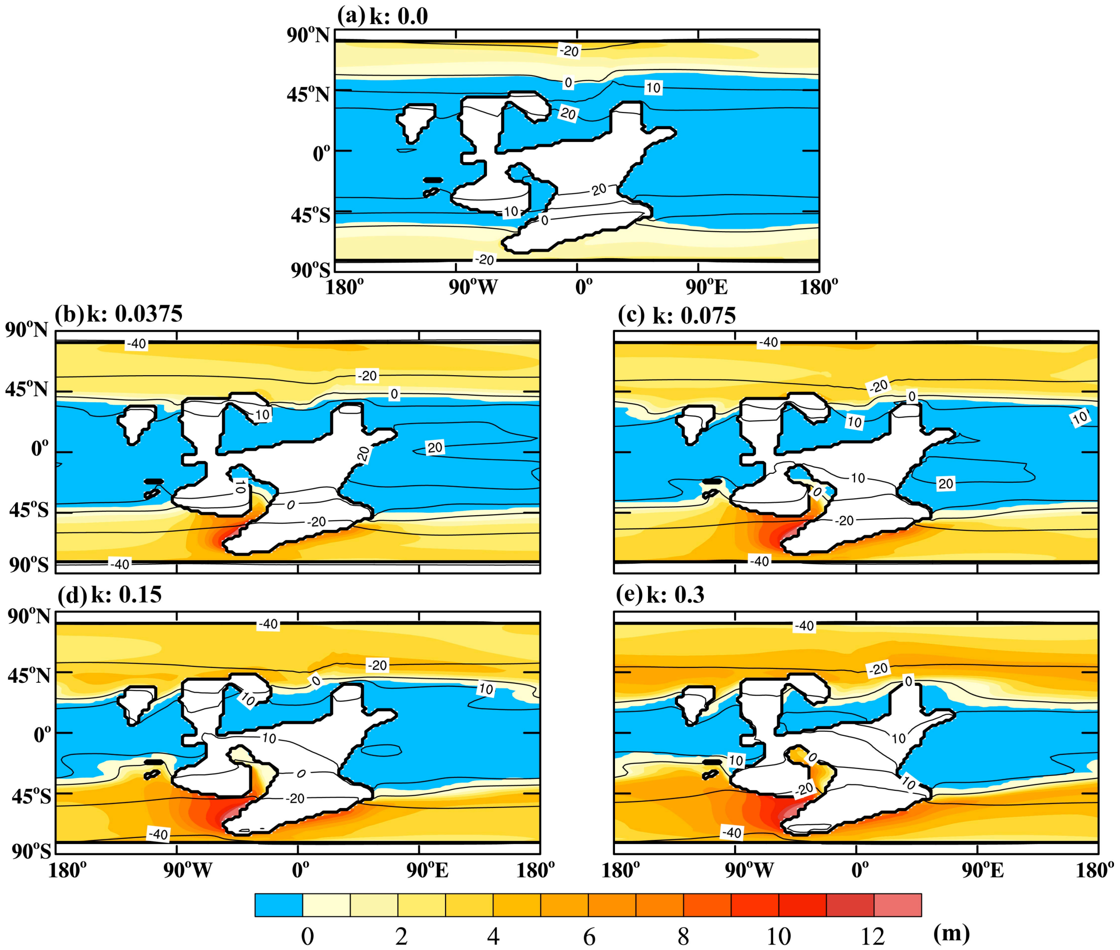

Supplementary Figure 7. Annual mean sea ice thickness (color) (Unit: m) and surface temperature (contour; Unit: °C) under different erodibilities when land vegetation is absent.

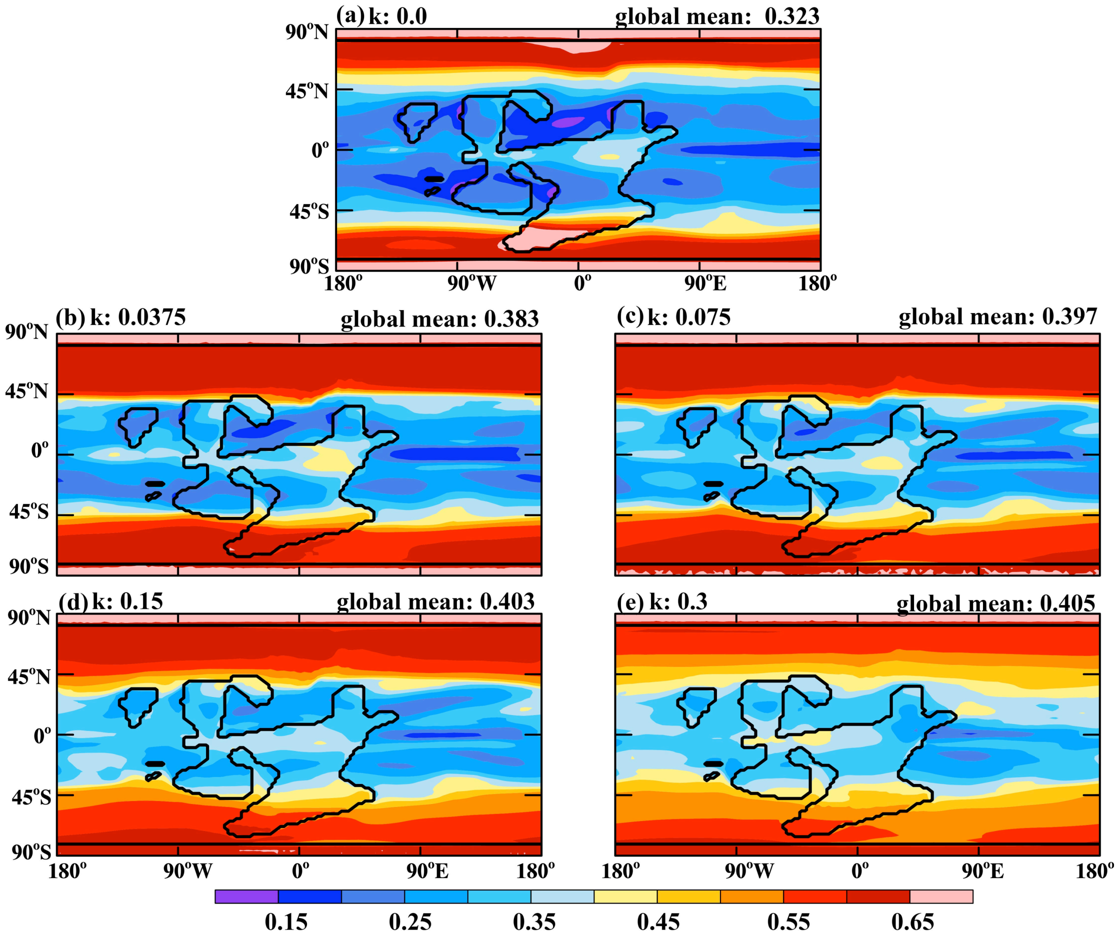

Supplementary Figure 8. Planetary albedo for different surface erodibilities when land vegetation is absent. Unit: %.

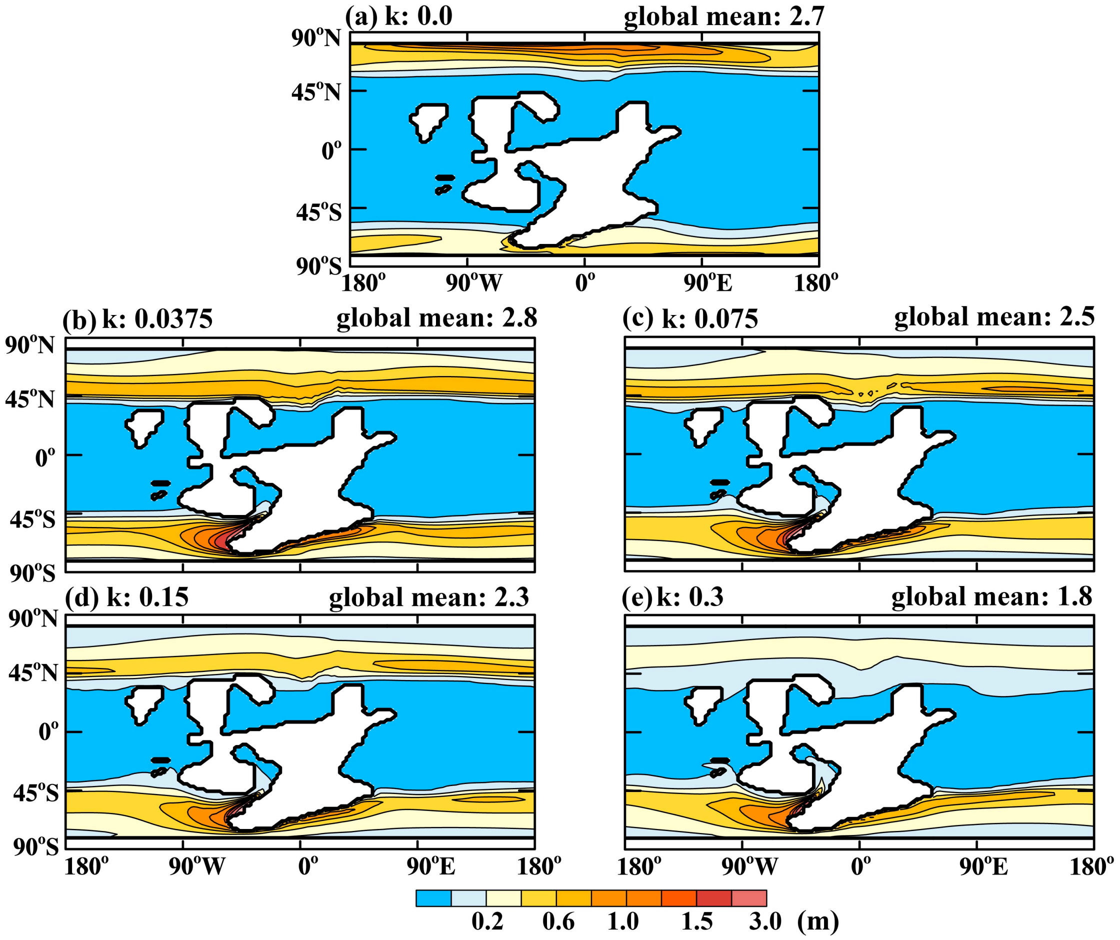

Supplementary Figure 9. Snow depth on sea ice under different erodibilities when land vegetation is absent. Unit: m.

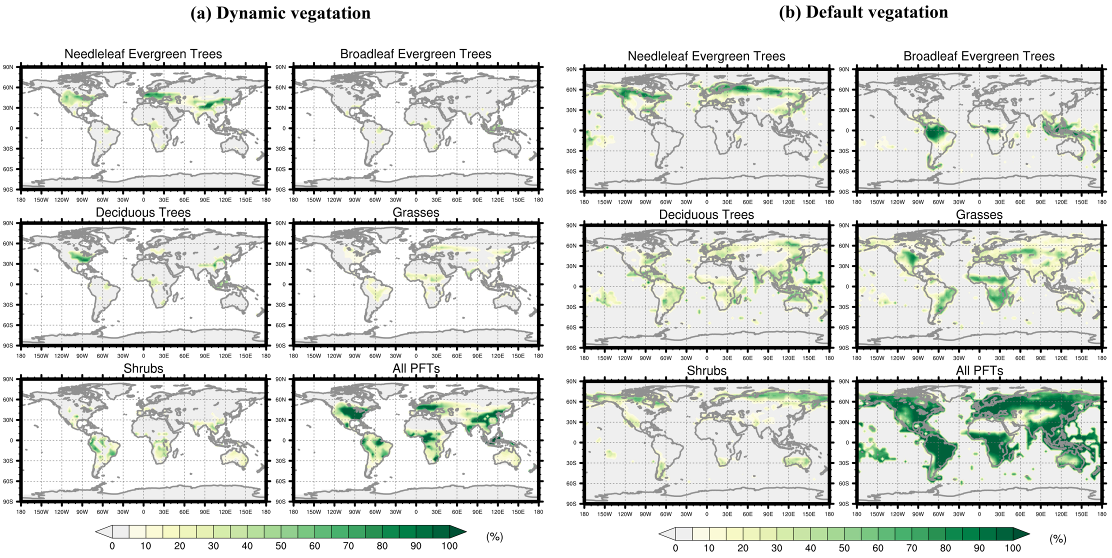

Supplementary Figure 10. Pre-industrial vegetation distribution. Panels (a) on the left show the concentration of representative vegetation types as well as the total vegetation (lower right panel) simulated by the dynamic vegetation scheme in the model starting from bare land (at model year 2600). Panels (b) on the right are the same except that these are the default vegetation distribution in the model set according to the observations. Unit: %. In this test, the model resolution is 3.75° × 3.75° (T31) for the atmosphere and land components, and ~1.6° (meridonal) × 3.6° (zonal direction) for the ocean and sea-ice components.

Supplementary References

1. Albani, S. *et al.* Improved dust representation in the Community Atmosphere Model. *Journal of Advances in Modeling Earth Systems* **6**, 541-570 (2014).

2. Bauer, S. E. & Menon, S. Aerosol direct, indirect, semidirect, and surface albedo effects from sector contributions based on the IPCC AR5 emissions for preindustrial and present-day conditions. *Journal of Geophysical Research: Atmospheres* **117,** D1 (2012).

3. Duce, R. *et al.* The atmospheric input of trace species to the world ocean. *Global biogeochemical cycles* **5**(3), 193-259 (1991).

4. Miller, R. & Tegen, I. Climate response to soil dust aerosols. *Journal of climate* **11**(12), 3247-3267 (1998).
